# Using electrocochleography to detect sensory and neural damages in a gerbil model

**DOI:** 10.1038/s41598-021-98658-6

**Published:** 2021-10-01

**Authors:** Sebastiaan W. F. Meenderink, Xiaohui Lin, Wei Dong

**Affiliations:** 1 VA Loma Linda Healthcare System, Loma Linda, CA 92374 USA; 2 Department of Otolaryngology, Head and Neck Surgery, Loma Linda University Health, Loma Linda, CA 92350 USA

**Keywords:** Neurophysiology, Physiology

## Abstract

Hearing is one of the five sensory organs that allows us to interact with society and our environment. However, one in eight Americans suffers from sensorineural hearing loss that is great enough to adversely impact their daily life. There is an urgent need to identify what part/degree of the auditory pathway (sensory or neural) is compromised so that appropriate treatment/intervention can be implemented. Single- or two-tone evoked potentials, the electrocochleography (eCochG), were measured along the auditory pathway, i.e., at the round window and remotely at the vertex, with simultaneous recordings of ear canal distortion product otoacoustic emissions. Sensory (cochlear) and neural components in the (remote-) eCochG responses showed distinct level- and frequency-dependent features allowing to be differentiated from each other. Specifically, the distortion products in the (remote-)eCochGs can precisely localize the sensory damage showing that they are effective to determine the sensory or neural damage along the auditory pathway.

## Introduction

Our hearing continuously provides us with acoustic information about our environment and plays a major role in our communication with other humans. Individuals are often unaware what the damage from blasts, noise, chemical exposure, and aging does to their hearing, but one in eight Americans suffers from some form of hearing loss that is great enough to adversely impact their daily life. Hearing loss often results from damage to sensory hair cells, but we currently lack efficient means to determine the etiology and intracochlear sites over which damage occurred.

Electrocochleography or eCochG, measures sound induced electrical potentials along the auditory pathway and typically is recorded differentially using a pair of electrodes. The position of these electrodes can vary considerably (e.g., silver-ball electrode on the round window or RW/promotorium, a tymp- or tip-trode in the ear canal, a scalp electrode on the mastoid), where the biggest concern seems a trade-off between the ease of electrode placement and the signal-to-noise ratio in the recordings^1,2^. The alternating current (AC) in the eCochG is a combination of the sensory response via the cochlear microphonics (CM)^3–5^ and the neural response, i.e., neurophonic^6–9^.

The sensory component of the eCochG, the CM, arises from the currents that flow through the mechano-electrical transduction (MET) channels primarily in outer hair cells (OHC)^4^. The CM seems to have poor spatial resolution as it is the (weighted) sum of potentials produced by all hair cells along the cochlear partition that are stimulated by the traveling wave along the basilar membrane (BM-TW)^10^. Moreover, the major contribution to tone-evoked CM is from OHCs in the base of the cochlea^11^ and less from mechano-sensory responses from the characteristic location (*CL: the location along the sensory epithelium requiring the smallest pure-tone input to evoke a threshold response. In the cochlea, this location systematically shifts to more apical locations with decreasing frequencies*). This is due to the phase characteristics of the sound-evoked BM-TW, which causes CM contributions from near the tone’s CL to cancel^12^. Often, the dominance of basal CM-sources over CL contributions is exacerbated by the placement of the recording electrode: when positioned near the base of the cochlea (e.g., at the RW), the closer proximity favors the contributions from the nearby, basally located, hair cells. However, location-specific information is available in the CM, and it can be extracted when the evaluated responses rely on CL-specific response properties of the cochlea. For example, Charaziak et al.^13^ demonstrated that CM responses can be used to detect and localize sensory hearing loss by exploiting the local, cochlear phenomenon of two-tone suppression.

The neural component in the eCochG reflects action potentials in auditory nerve fibers that synchronized to the stimulus, both at the onset (compound action potentials; CAP) and during the remainder of the stimulus. The latter is due to a phenomenon called phase-locking in which action potentials occur during a preferred phase of the response-evoking periodic stimulus^8,9^. Neural phase-locking is a fundamental property of neurons and has been proposed to be critical for many aspects of hearing (see review^14,15^). Phase-locking is a low-frequency phenomenon: in auditory nerve fibers it gradually decreases for frequencies above ~ 1 kHz and is absent beyond ~ 5 kHz (i.e.,^9,14,15^).

The combined nature of eCochG^7,16^ makes that it holds information on the health of both the sensory and neural parts of the auditory system, although its mixed nature also complicates its clinical utility as a diagnostic tool.

In the current study we use the eCochG to differentiate between sensory and neural hearing loss. We simultaneously recorded eCochG in gerbils with two electrode pairs. The first pair had the active electrode “far-removed” from the cochlea, i.e., subcutaneous at the vertex of the skull, which we refer to a remote-eCochG. The active electrode for the second electrode pair was placed at the edge of the RW antrum at the base of the cochlea (termed “RW-eCochG” from here on). Simultaneously with eCochG, ear-canal sound pressure was measured close to the tympanic membrane. We demonstrate the level- and frequency-dependent characteristics of the cochlear (sensory) and neural components in eCochG and discuss how these can be used to differentiate between sensory and neural hearing loss. Further, we illustrate how changes in two-tone induced distortion products (DPs) in the eCochGs detect local sensory damage in the cochlea, in a similar manner as distortion product otoacoustic emissions (DPOAEs). These results show how eCochG can be used to detect sensory or neural defects in the auditory periphery.

## Results

**Figure 1:**
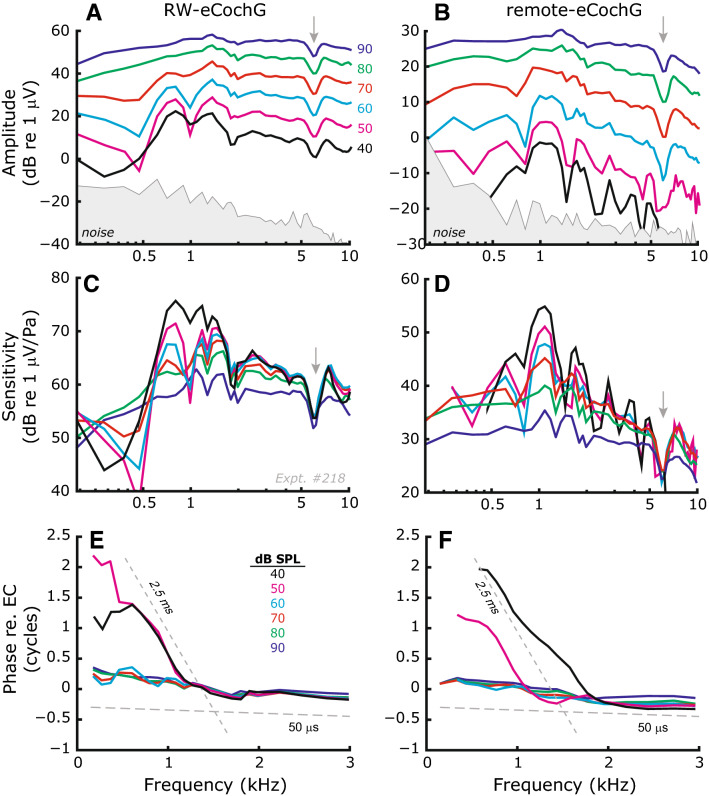
RW-eCochG and remote-eCochG in normal ears. Amplitude, sensitivity, and phase responses of eCochG measured with an electrode at the round window (**A**, **C**, **E**) and at the vertex of the skull (**B**, **D**, **F**), respectively. Sound pressure levels (SPL) were between 40 and 90 dB SPL in 10 dB steps. Sensitivity curves (amplitude normalized to ear canal pressure) would overlie each other if amplitude increased linearly with SPL. Dashed lines in panels (**E**, **F**) indicate group delays of 2.5 and 0.05 ms, respectively. Data from Expt. #218. The notch around 6 kHz (*gray arrows*) results from particulars in gerbil middle ear transmission.

**Figure 2:**
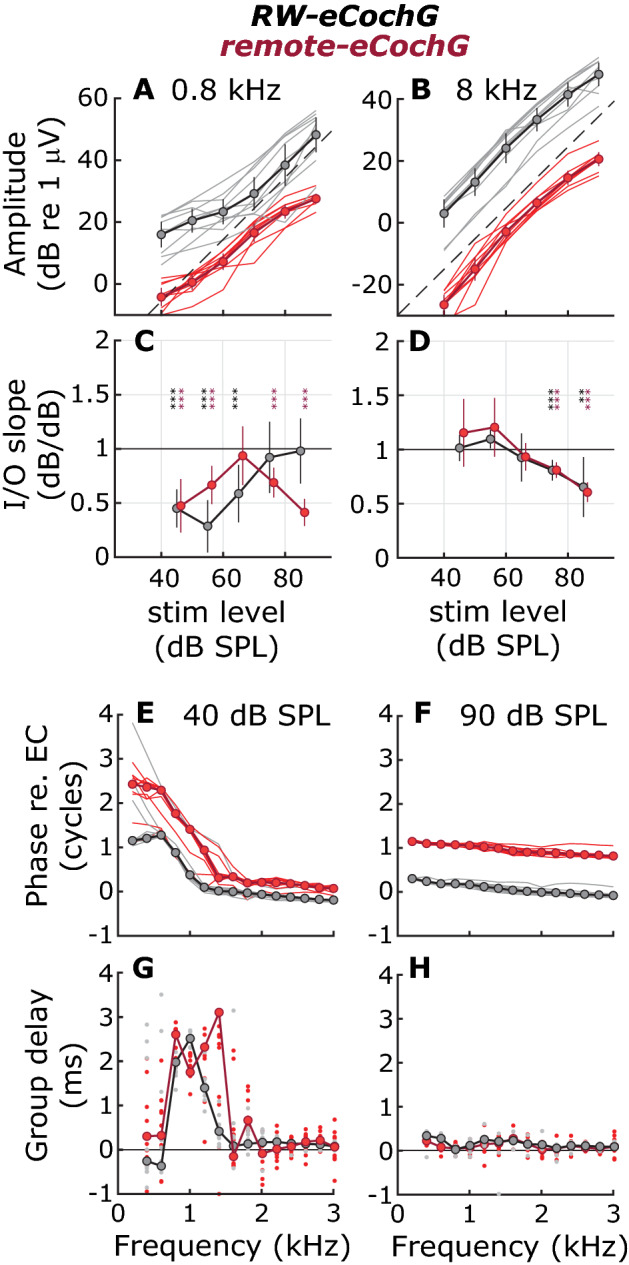
Level dependence, phase, and group delays for RW-eCochG and remote-eCochG in normal ears. (**A**, **B**) Input and output (I/O) curves of RW-eCochG (*black*) and remote-eCochG (*red*) amplitude in n = 10 animals at two stimulus frequencies. Error bars give 95% confidence intervals around the mean (*circles*). The slopes of these I/O curves are shown in (**C**, **D**), where a slope of 1 dB/dB and < 1 dB/dB represents linear and compressive nonlinear growth with SPL, respectively. At each stimulus level, a t-test was used to assess whether the slope was significantly smaller than unity. *Asterisks* indicate the probability with:    **p < 0.01; ***p < 0.005. A small horizontal offset between RW- and remote-eCochG responses was introduced for clarity. (**E**, **F**) Phase-vs-frequency curves for the same n = 10 animals at two different stimulus levels. (**G**, **H**) Corresponding group delays. Both individual (*thin lines/small dots*) and average (*circles*) are shown.

**Figure 3:**
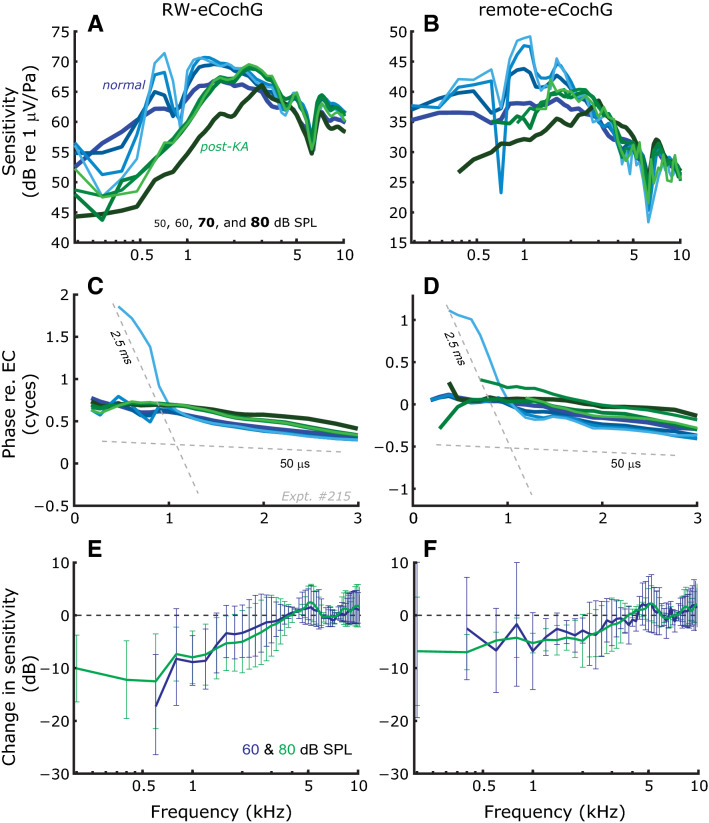
Effects of kainic acid (KA) on RW- and remote-eCochG. Representative examples (Expt. #215) of eCochG sensitivity (**A**, **B**) and phase (**C**, **D**) before (*blue lines*) and after (*green lines*) the application of KA to the round window antrum. *Dashed lines* in panels (**C**, **D**) indicate delays of 2.5 and 0.05 ms, respectively. (**E**, **F**) Average change in eCochG sensitivity (n = 5 animals) after KA application at stimulus levels of 60 (*blue lines*) and 80 dB SPL (*green lines*), respectively. Error bars denote ± 1 standard deviation (s.d.).

**Figure 4:**
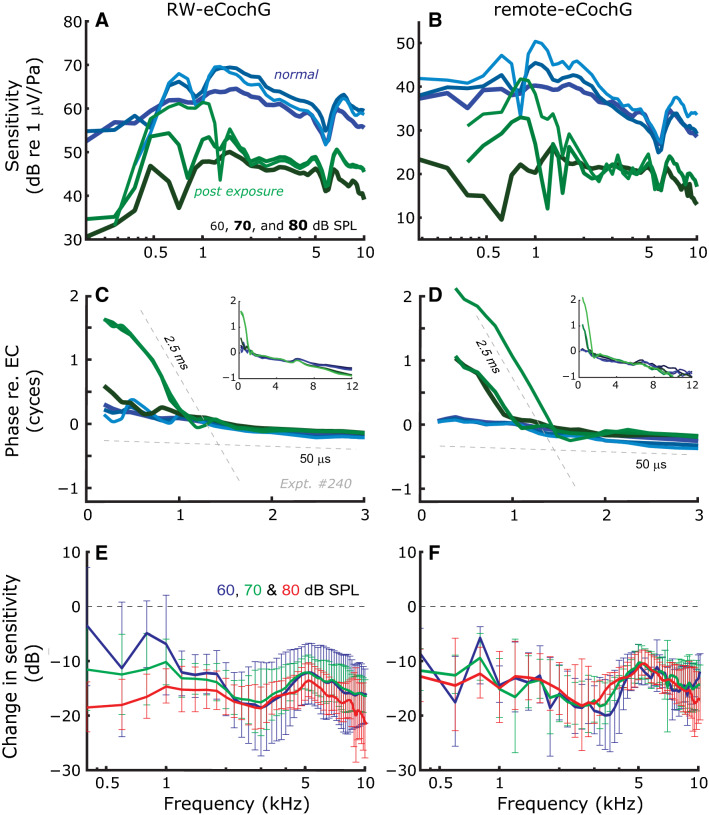
Effects of exposure to intense, broadband sound on RW- and remote-eCochG. Representative examples (Expt. #240) of eCochG sensitivity (**A**, **B**) and phase (**C**, **D**) before (*blue lines*) and after (*green lines*) exposure to intense (110 dB SPL, 15-20 min), broadband sound. *Dashed lines* in panels (**C**, **D**) indicate delays of 2.5 and 0.05 ms, respectively. The insets show phase data over an extended frequency range. (**E**, **F**) Average change in eCochG sensitivity (n = 5 animals) after exposure for stimulus levels of 60 (*blue lines*), 70 (*green lines*) and 80 dB SPL (*red lines*), respectively. Error bars denote ± 1 s.d.

**Figure 5:**
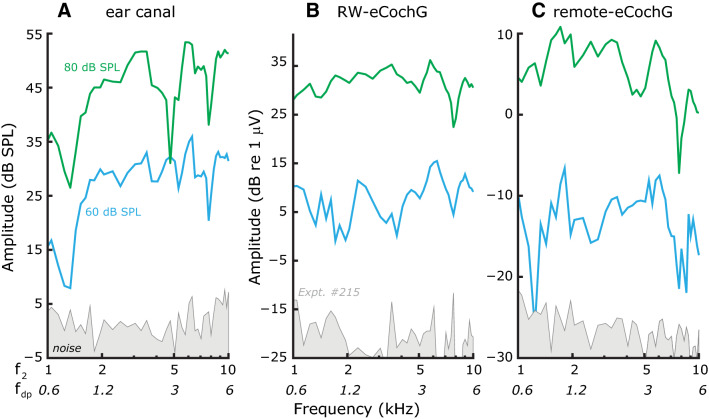
Distortion products (DP) in normal ears. Representative examples (Expt. #215) of DPs in (**A**) ear canal sound pressure (DPOAEs), (**B**) RW-eCochG, and (**C**) remote-eCochG. The three measures were taken simultaneously. DPs were evoked by presenting two stimulus tones that had their frequency ratio f_2_/f_1_ fixed at 1.25. The levels for the two stimulus tones were equal at L_1_ = L_2_ = 60 (*blue lines*) or 80 dB SPL (*green lines*), respectively.

**Figure 6:**
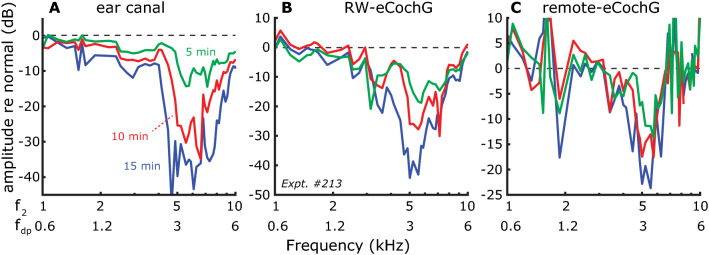
Cumulative effect of exposure to an intense 4-kHz tone on distortion products. Representative examples (Expt. #213) of changes in DP amplitudes in (**A**) ear canal sound pressure, (**B**) RW-eCochG, and (**C**) remote-eCochG following progressively longer exposure to a 100-dB tone at 4 kHz (*colored lines*). DPs were evoked by presenting two stimulus tones (with L_1_ = L_2_ = 60 dB SPL) using a fixed frequency ratio f_2_/f_1_ = 1.25. Data from this experiment are included in the averaged effects shown in Fig. 7.

**Figure 7:**
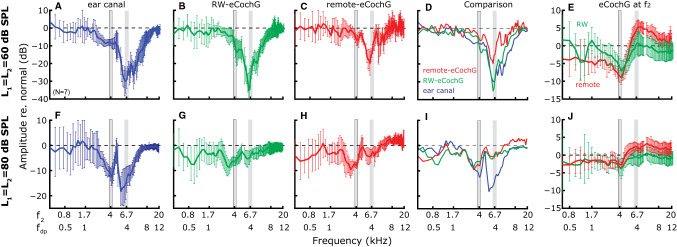
Effects of exposure to an intense, 4-kHz tone on RW- and remote-eCochG. (**A**–**C**) Average (± 1 s.d.) change in DP amplitude after exposure to an intense, 4-kHz tone (100 dB SPL, 15 min). DPs were evoked with L_1_ = L_2_ = 60 dB SPL and f_2_/f_1_ = 1.25, and simultaneously measured as (**A**) DPOAEs in the ear canal and as DPs in (**B**) RW-eCochG and (**C**) remote-eCochG. Mean DP changes from A–C are superimposed in (**D**) to aid their comparison. Vertical *gray bars* indicate 4 kHz, either as stimulus, i.e., f_2_ or DP frequency. (**E**) Averaged (± 1 s.d.) change in eCochG amplitudes for the f_2_ stimulus tone. The *green* and *red line* are for RW- and remote-eCochG, respectively. (**F**–**J**) Same layout as (**A**–**E**), but for L_1_ = L_2_ = 80 dB SPL.

**Figure 8:**
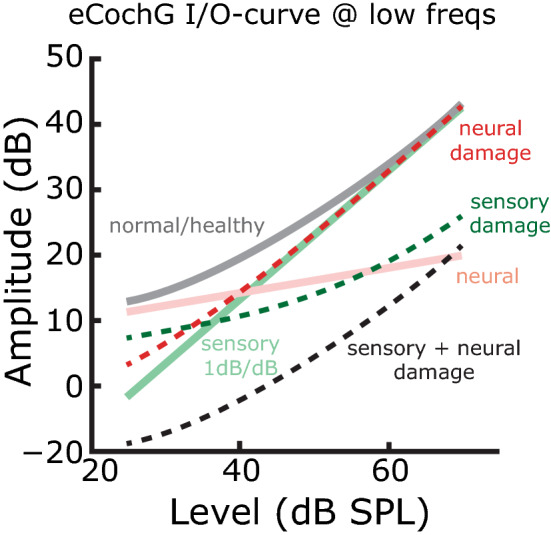
Detection of sensory or neural damages using low frequency, eCochG input/output (I/O) curves. The eCochG I/O curve (*gray*) is composed of linear sensory (*solid green*, 1 dB/dB) and compressive nonlinear neural component (*solid pink*, < 1 dB/dB). Damage to the sensory and/or neural component results in different changes of the I/O-curves (*dashed lines*), providing a means to identify different types of hearing loss.

We first used responses to single tones to confirm the dual origin (sensory and neural) of the EcochG and show that these separate responses can readily be identified by evaluating either their level-dependence and/or their delays (Figs. 1, 2, 3, 4). These data also illustrate that the recording-electrode configuration has only marginal effects on the obtained results. Second, we provide data in which two-tone evoked DP components in the eCochG identify location-specific sensory (cochlear) damage. These results are compared to simultaneously measured ear-canal DPOAEs (Figs. 5, 6, 7). We demonstrate the AC component of the eCochG can be used as a diagnostic tool to differentiate sensory and neural hearing loss (Fig. 8). Across animals, the eCochG responses were similar under similar modifications along the auditory pathway. In each section, we use representative responses from an individual animal to demonstrate our main findings, followed by statistical analysis across animals.

### Characterization of the sensory and neural component in eCochG

#### ECochG dependency on level and frequency

Figure 1 shows RW-eCochG and remote-eCochG for a representative animal in response to single tones that were presented in the ear canal over a range of frequencies and levels. Although the remote-eCochG responses are ~ 20-dB smaller and are moderately low-pass filtered beyond 7.5 kHz (see “Materials and methods”), both metrics are qualitatively similar. Below 2 kHz, the amplitudes of the responses show pronounced fine structure for low to intermediate stimulus intensities (Fig. 1A, B) and grow compressively (Figs. 1C, D and 2A–D), while the corresponding phase-vs-frequency curves have a steep slope with a group delay (defined as $\tau =-\frac{\nabla \varphi }{\nabla f}$) of about 2.5 ms (Figs. 1E, F and 2E–H). At higher sound intensities the fine structure diminishes, and the phase dependence on frequency becomes less pronounced (group delay < 0.1 ms). For frequencies above 2 kHz, response characteristics at all stimulus levels are like the high-level responses observed below 2 kHz: amplitude varies smoothly with stimulus frequency and grows almost linearly at 1 dB/dB with intensity (some compression is evident at 90 dB SPL because of saturation of MET channels), while the phase-vs-frequency curves exhibit only little group delay. Similar results were obtained from all animals tested (also see blue lines in Figs. 3, 4). The similarities between RW- and remote-eCochG are consistent with previous observations^9,17^ and confirms that the latter is a far-field representation of the responses recorded near the RW.

#### Sensory component of the eCochG

To confirm the dual nature of the eCochG, we obtained recordings following the application of kainic acid (KA) to the round window (Fig. 3). This neurotoxin has an excitotoxic effect on afferent auditory neurons, essentially abolishing the action potentials in the auditory nerve. The KA application (Fig. 3, *green lines*) only affected the eCochG for low-frequency stimuli (< 2 kHz) with similar effects across animals (Fig. 3E, F). The eCochG sensitivity no longer varied with intensity and the fine structure was lost (Fig. 3A, B). It also removed the long latency components (group delay > 2 ms) that are typically observed for low to intermediate stimulus intensities (Fig. 3C, D). What remained in the eCochG was the sensory component, which resembles a high-pass-filtered response that grows linearly at 1 dB/dB with stimulus level and has a small group delay.

#### Neural component of the eCochG

Next, the sensory component within the eCochG was reduced by about 20 dB by exposing animals for 15–20 min to intense, broadband sound of 110 dB SPL immediately prior to the recordings (Fig. 4). For higher stimulus frequencies (> 2 kHz), the overexposure resulted in substantial reduction of eCochG sensitivities (Fig. 4A, B; *green lines*), and a small but systematic effect on (the slopes of) the phase responses (Fig. 4C, D *insets*). These slightly longer group delays suggest that the sustained sensory damage was most severe near the base of the cochlea such that the remaining sensory component primarily arose from hair cells at more apical cochlear locations (re. normal). For frequencies below 2 kHz, the effect of overexposure depended on the intensity of the stimulus. For the highest SPL, responses were similarly reduced as the high-frequency responses in all animals tested (Fig. 4E, F). Combined with the shallow phase-vs-frequency curves this indicates that the sensory component, although substantially reduced, still dominated the overall eCochG response. For low and intermediate stimulus intensities a different picture emerged. Here, the post-exposure (*green lines*), response amplitudes were bandpass shaped, grew compressively with increasing SPL, and phase exhibited large group delays, which were absent pre-exposure (*blue lines*) at these stimulus levels (See also Figs. 1, 2). Note that a reduced sensory response likely also reduced the neural response, as the latter is a consequence of the former. However, the neural response will be affected less than the CM itself due to its compressive level-dependence.

Consistent with literature^9,17^, these data show that the AC-component in the eCochG combines sensory and neural components, each one with distinct frequency- and level-dependent characteristics. Only for low frequencies (< 2 kHz) and low intensities (< 50 dB SPL) may the phase-locked neural contribution exceed the sensory component, which can be easily identified by the long group delay (> 2 ms) and/or the compressive level-dependence of the response. At higher frequencies the eCochG is dominated by the sensory component that increases linearly with sound pressure levels and shows a short group delay, suggesting it originates from hair cells near the base of the cochlea.

### Using the two-tone induced DP component in eCochG to detect location specific sensory damage

In response to a two-tone stimulus, the healthy cochlea generates DPs because of the nonlinear OHC transduction process. These DPs can be recorded in the ear-canal sound pressure as DPOAEs (Fig. 5A) but are also well above the estimated noise floors in the two eCochG recordings (referred to as eCochG-DPs; Fig. 5B, C). Since the generation of intracochlear DPs and DPOAEs is associated with normal OHC functioning it can be used to detect sensory hearing loss.

To illustrate, Fig. 6 shows the effects of progressively longer exposure to an intense 4-kHz tone at 100 dB SPL on the DPOAEs (Fig. 6A) and eCochG-DPs (Fig. 6B, C). In this example, the initial 5-min exposure (*green lines*) caused a frequency dependent reduction in DP amplitude in all three recording channels, with the largest reductions around f_2_ = 5–6 kHz (corresponding to 2f_1_ − f_2_ ≈ 3–4 kHz). This is consistent with the notion that DPs are primarily from the nonlinear distortion region, basal and close to the f_2_ peak^18^. Subsequent exposures (*red* and *blue lines*) increase this initial effect, resulting in more substantial reductions of the DPs at f_2_ = 5–6 kHz. These data also suggest that the frequency range over which DPOAEs are affected is wider re. that for the eCochG-DPs.

We obtained similar two-tone (L_1_ = L_2_ = 60 dB SPL; f_2_/f_1_ = 1.25) recordings from n = 7 gerbils that were exposed to 10–15 min of this intense, 4-kHz tone (Fig. 7A–E). The RW-eCochG responses at stimulus frequency f_2_ were reduced, but only moderately (– 5 dB) by the sustained damage (Fig. 7E; *green line*). Changes for the f_1_ stimulus tone were similar and are not shown. The effect was larger in the remote-eCochG with a maximum reduction of 10 dB (Fig. 7E; *red line.* Again, f_2_ and f_1_ responses were identical). This suggests that CM is not exclusively from the cochlear base; CL does contribute to the CM response. Besides the reduction for stimulus frequencies below 4 kHz, there is now significant enhancement of the responses around 4 kHz, especially in the remote-eCochG. This is explained by considering that the CM is the vector sum of receptor currents from all hair cells that are stimulated along the cochlear partition by the BM-TW. Due to the BM-TW phase characteristics, basally originating CM components are partially cancelled by CM components from CL under normal conditions. The damage around CL reduces the components from this region, effectively reducing their “suppressive” effects on the overall CM response.

Although the effects of the induced sensory damage were moderate on the responses to the stimulus tones, they had a pronounced effect on the measured DPs. The DPOAEs, i.e., DPs in the ear canal (Fig. 7A), were reduced over a wide frequency range (1–10 kHz) with the most substantial reductions for 2f_1_ – f_2_ = 4 kHz of 20–30 dB. The DPs in RW-eCochG (Fig. 7B) and remote-eCochG (Fig. 7C) were also affected in a frequency specific way; they showed a substantial decrease when 2f_1_ − f_2_ was centered around the 4-kHz, notch-frequency, while both lower- and higher-frequency eCochG-DPs were less affected by the induced damage. A comparison of the three DP measures (Fig. 7D) reveals that the frequency specificity to detect the notch is highest in remote-eCochG, followed by the RW-eCochG-DPs and DPOAEs, respectively. This shows that eCochG-DPs can readily be used to inform about location-specific sensory damage in the cochlea.

When using higher-intensity stimuli (L_1_ = L_2_ = 80 dB SPL), a different picture emerges (Fig. 7F–J). First, there is less reduction in DP amplitudes for all three measurements. Second, the DPOAE reductions are now bimodal (Fig. 7F) with local minima around 2f_1_-f_2_ = 2 kHz (f_2_ = 3–4 kHz) and 4 kHz (f_2_ = 5–6 kHz), respectively. The change in eCochG-DP amplitudes (Fig. 7G, H) also show this bimodal dependence on frequency, but less pronounced: maximum reductions occur for 2f_1_ − f_2_≈2 kHz. The origin of this bimodal dependence is not obvious. It could arise from the selective abolishment of distortion-type and reflection-type DP components^18^ but may also result from the interference of “CL” and “sub-CL” DP components. The latter would explain why the bimodal dependence is not as pronounced for L_1_ = L_2_ = 60 dB SPL, since “sub-CL” components become more important with increasing stimulus levels. At this higher intensity, the responses to the stimulus themselves are not affected by the sensory damage (Fig. 7J). Apparently, they are now dominated by OHC transduction currents in the base of the cochlea, with no significant contributions from more apical locations.

## Discussion

We presented recordings of remote-eCochGs, RW-eCochGs and the ear canal pressure, all in response to single and two-tone stimuli. By exposing the cochlea to KA (Fig. 3) or intense, broadband sounds (Fig. 4) we confirmed the notion that eCochG combines a sensory (i.e., CM) and neural response that differ in their frequency and level-dependence. In general, the data show that the AC-component in the eCochG is dominated by a linear sensory component with a short group delay; only for low frequencies (< 2 kHz) and low intensities (< 50 dB SPL) may the phase-locked neural contribution exceed the sensory component. This, however, is easily identified by the long group delay (> 2 ms) and/or the compressive level-dependence of the response.

The short group delays (~0.05 ms) associated with the sensory (CM) component indicate that the eCochG primarily originates from OHC near the base of the cochlea. For RW-eCochG, this could be due to the position of the recording electrode, which favors responses from nearby hair cells (i.e. those in the base of the cochlea). But the phase group delays were similar in the remote-eCochG, in which case all OHCs are (almost) equally far away from the recording electrode. Rather than caused by the electrode placement, it seems that the sensory component of the eCochGs is dominated by signals from basal OHCs due to the traveling wave properties in the cochlea. Locally measured CM near the cochlear partition show similar tuning and phase characteristics as the BM-TW^19,20^: in the basal region of the cochlea, the transduction currents are all in phase and thus add up, while contributions from cells further away (towards the apex), i.e., CL, tend to cancel each other because of the quickly varying phase.

We found group delays for the neural component between 2.3 and 2.5 ms. This short delay excludes contributions from auditory brainstem nuclei: the measured neural response originates from the auditory nerve. Assuming a synaptic delay of 1 ms^21^, the remaining 1.3–1.5 ms in this delay is mostly from cochlear travel time, which suggests that the signals originate from nerves that innervate a more apical region of the cochlea, perhaps the frequency’s tonotopic location (CL). However, based on ANF responses in gerbil^22^, a 1-kHz tone would take longer than 1.5 ms to propagate to its tonotopic location (on average 2.2 ms). Rather, the observed delays suggest that the major contributors are nerve fibers that innervate hair cells at a location with a CF approximately 1 octave higher than the stimulus. Again, this is probably related to the speed at which waves propagate in the cochlea, which causes responses from around CL to cancel. In contrast to the CM, the major contribution is not from the extreme cochlear base because the neural responses to low-frequency tones are too weak there. Although combining constructively (in-phase), the sum of these smaller contributions does not exceed the sum of the almost-in-phase, but individually larger, responses from a cochlear location that is between the cochlear base and CL. In summary, the CM evaluated at the stimulus frequencies is a location-specific response. However, this location is near the base of the cochlea and is therefore unrelated to the health status at a tone’s CL for all but the highest frequencies.

Notwithstanding, eCochG responses to single tones can in principle be used to assess (high-frequency or broadband) sensory or neural hearing loss by considering their level-dependence in response to low-frequency stimuli (Fig. 8). That is, under normal, healthy cochlear conditions the low frequency, eCochG input/output or I/O-curve consists of two regions that exhibit different growth rates. For intensities below ~ 50 dB SPL, growth will be compressive (< 1 dB/dB; “shallow”) because the neural component dominates, while for higher intensities the slope of the curve will be 1 dB/dB (“steep”) because the CM is the largest component in the response. A reduction in the neural component (i.e., neural hearing loss) effectively extends the intensity range over which the CM dominates, and the transition from a “shallow” to a “steep” I/O-curve shifts to lower stimulus intensities. At higher intensities, no changes in eCochG are expected for neural hearing loss. Sensory hearing loss will cause a reduction in both the CM and neural component in the eCochG, but this reduction is relatively larger for CM. Consequently, the entire I/O-curve appears shifted vertically to lower intensities *but* it will exhibit a more pronounced, low-intensity shallow tail. The combination of sensory and neural hearing loss will combine these two effects: a vertical shift towards lower levels, combined with a less pronounced or absent shallow low-intensity tail.

Due to the heavy involvement of basal OHC, this method is not ideally suited to identify frequency/location-specific sensory hearing loss, although responses to the stimulus tones are reduced by the induced damage in a frequency specific way (Fig. 7). To extract more precise frequency specific information about sensory hearing loss from the eCochG, it is better to consider not the responses to the stimulus tones directly, but to use them to evoke a nonlinear cochlear response that is location specific, related to the stimulus CL. This strategy was demonstrated^13^ by exploiting the phenomenon of cochlear two-tone suppression to identify and localize cochlear deficiencies from the RW-eCochG. Here we used another nonlinear, cochlear phenomenon, the generation of DPs (Figs. 6, 7). The premise is that the eCochG-DPs arise from a restricted spatial region, predominantly from a cochlear region associated with the f_2_ CL. This localized DP-origin is supported both by experiments and model simulations, at least when applied to ear-canal DPOAEs^18,23^. Although all OHCs that are under the entire f_2_ envelope (i.e., from base to f_2_ CL) will produce DPs^24^, apparently those DP components from around the f_2_ CL contribute significantly to both the ear-canal DPOAE or the eCochG-DP. What limits this spatial extent seems to be the location-dependent properties of the BM traveling wave. Its variable speed causes cancellation of DP components along most of the cochlear partition, while its variable amplitude further emphasizes contributions from (just basal of) f_2_ CL.

The origins of eCochG-DPs and DPOAEs are, however, not necessarily the same. The dissimilarity between the DPOAE and eCochG-DP responses evoked with 80 dB SPL (Fig. 7) indeed suggests that they originate from different (extents of) cochlear regions at these high stimulus intensities. This difference may relate to the existence of a potential secondary cochlear source for eCochG-DPs that is different from the nonlinear distortion generation mechanism^25^. These “secondary” eCochG-DPs are OHC responses that are driven by the “primary” DPs that propagate in both the basal and apical direction after generation. The basal, secondary eCochG-DP sources are more pronounced at high stimulus intensities, and thus contribute significantly to the overall eCochG-DP. For lower intensities, the contributions from the secondary eCochG-DP sources are much reduced, rendering the (cochlear origins of) DPOAEs and eCochG-DPs more similar. It is the location-specific origin of the eCochG-DPs (at moderate stimulus levels) that allows them to locate CL sensory hearing loss (Fig. 7). Compared to DPOAE measurements, their specificity appears higher, which is probably related to a more restricted cochlear extent (re. DPOAEs) that is involved in their generation. Although the eCochG-DPs can readily identify the cochlear location, we made no attempts to also determine the severity of the sensory hearing loss. The eCochG-DP data obtained during progressive exposure to intense sounds (Fig. 6), however, strongly suggest that this information is available in the amplitudes of the eCochG-DPs. More extensive studies that characterize eCochG responses together with the extent and severity of hearing loss are needed to further establish their relationship. Even so, we showed that eCochG responses, even when recorded with electrodes that are “far away” from the cochlea (e.g., skin-surface electrodes as typically used during auditory brainstem or ABR recordings), can be used to identify neural and/or sensory neural hearing loss. For the latter, location-specific information about the damage can be obtained by utilizing the nonlinear cochlear response (in the form of DP-generation). Implementation of these techniques in the clinic is feasible, given the ubiquitous presence of ABR-related hardware, and should provide a sensitive tool to diagnose different types of (peripheral) hearing loss.

## Materials and methods

### Animal preparation

The care and use of animals were approved by the Institutional Animal Care and Use Committee (IACUC) of the VA Loma Linda Healthcare System. The study was carried out in compliance with the ARRIVE guidelines and all methods were carried out in accordance with relevant guidelines and regulations. We present data from 16 young adult healthy Mongolian gerbils (*M. unguiculatus*; 40–75 g body weight) in the current study. An additional nine gerbils were used for preliminary data acquisition, and to finalize recording protocols. Gerbils were deeply anesthetized by an intraperitoneal injection of ketamine/xylazine (80 and 20 mg/kg, respectively) with maintenance doses (at 1/3 of the initial dose) given when needed to maintain areflexia throughout the experiment. Buprenex (0.2 mg/kg) was injected every six hours (intraperitoneally) and saline solution was periodically administered subcutaneously to keep the animal hydrated. Once the animal was deeply anesthetized, its head was fixed to minimize motion, and a tracheotomy was performed to ensure a patent airway. No active ventilation was used. The left pinna and cartilaginous ear canal were removed, and a custom-built speaker/microphone probe system was sealed to the exposed bony ear canal. The ipsilateral bulla was widely opened to allow access to the round window antrum. Cochlear condition was assessed at regular intervals using DPOAEs. Rectal temperature was maintained at ~ 37 °C.

### Experimental setup

Experiments were performed in a sound-attenuating booth, with the animal placed on a vibration-isolation table. Acoustic stimuli were composed of either one or two frequency components. Frequency components were generated from separate D/A channels (Tucker–Davis Technologies or TDT RX6, F_sam_ = 200 kHz) and speakers (Fostex), and chosen such that they went through an integer number of cycles over 4096 samples (~ 21 ms). Waveforms were presented for 52 × 4096 (for SPL > 70 dB SPL) or 402 × 4096 samples (for SPL < 70 dB SPL) and included 5-ms ramps (raised cosine) at their on- and offset. To exclude these ramps from the analysis, only the center 50 × 4096 or 400 × 4096 samples were further analyzed. This separation of frequency components minimizes system distortion during two-tone stimulation. The speakers, which were shielded and grounded by enveloping them in multiple layers of aluminum foil, were connected to the custom-built probe system via plastic tubing. The probe system also contained an ultrasound probe-tube microphone (Sokolich, 1 V/Pa sensitivity) which had its tip positioned within 3 mm from the tympanic membrane. The microphone signal was digitized and used for in situ speaker calibration and recording of ear-canal sound pressure (ECP) during the experiments. Besides ECP, we simultaneously measured the eCochG using two pairs of electrodes. The first pair consisted of a silver-ball electrode on the promotorium near the round window (RW) antrum and a platinum reference electrode in one of the surgically exposed muscles in the neck. The signals from these electrodes were differentially amplified and bandpass-filtered (SRS560; 1000x, 0.1–30 kHz) prior to A/D conversion. We refer to these recordings as RW-eCochG throughout the manuscript. The second electrode pair consisted of two subdermal stainless-steel electrodes, placed at the vertex of the skull (active) and over the ipsilateral bulla (reference), respectively. They were differentially amplified by a low impedance headstage (TDT RA4LI; 20x) that was connected to a preamplifier (TDT RA4PA). The latter provided additional amplification (250x) and low-pass filtering (F_corner_ = 7.5 kHz; 6 dB/oct roll-off) prior to A/D conversion (F_sam_ = 25 kHz). These recordings are  referred to as remote-eCochG. A subdermal electrode at the ankle of one of the hind limbs served as a common ground. All electrode impedances were ~ 1 kΩ. Stimulus generation and data acquisition were controlled by custom software written in Matlab.

### Data analysis

Digitized waveforms for ECP, RW-eCochG, and remote-eCochG were stored on computer disk for subsequent analysis in Matlab. The amplitude, phase, and the associated noise floor at the desired frequencies (i.e., stimulus and intermodulation frequencies) were extracted using Fourier analysis. The reported phase of the eCochG responses is relative to the ECP phase, while sensitivity of the response was calculated as the amplitude ratio of eCochG to ECP.

### Suppression of the neural eCochG component

In five animals we used kainic acid (KA; Abcam) to suppress the neural contributions to the eCochG recordings. KA is an analog of l-glutamate that has an excitotoxic effect on afferent auditory neurons^26^. The KA diminishes neural activities by removing excitatory postsynaptic potential evoked by the hair cell neurotransmitter^27^. The KA was delivered to the cochlea passively via diffusion through the round window. It was dissolved in Lactated Ringer to a molar concentration of 60 mM, which was used to fluid-fill the RW niche for 1 h. Recordings were obtained after removal of the fluid from the RW niche. As a control we applied Lactated Ringer alone, which had no discernible effects on the eCochG recordings post-exposure.

### Suppression of the sensory eCochG component

Two groups of animals were used to explore the effects of broadband or local sensory trauma, respectively. Broadband trauma was induced by exposure to a sequential series of intense 4096 (~ 21 ms) tones (frequencies: 0.2–50 kHz; 110 dB SPL) that were continuously presented for 15–20 min. Notch-type damage came from exposure to a continuous intense (100 dB SPL) single tone at 4 kHz. Here, the tone was presented in a few iterations (5 min/iteration), which allowed us to follow the progression of the sustained damage with accumulating overexposure.
